# Analysis of gas–solid two-phase flow and structure optimization of mobile shot blasting machine recovery device

**DOI:** 10.1038/s41598-022-26481-8

**Published:** 2022-12-23

**Authors:** Yinhang Zhang, Xiuli Fu, Xiuhua Men, Yongzhi Pan, Tianyi Zhang, Zhenfeng Jiang

**Affiliations:** 1 School of Mechanical Engineering, University of Jinan, Jinan, 250022 China; 2 Zibo Daya Metal Technology Co., Ltd. Zibo, Shandong, China

**Keywords:** Engineering, Mechanical engineering

## Abstract

To address the problem of low efficiency of recycling process waste by gas–solid two-phase flow of the shot blasting machine recycling device, a method and structure by increasing the negative pressure value and optimizing the outlet pipe position are proposed. Computational fluid dynamics (CFD), discrete element method (DEM) and discrete phase model (DPM) were used to study the waste recovery efficiency at different pressure outlet conditions and outlet pipe locations. The validity of the model was verified by velocity tests at the outlet and inlet compared with simulations. The effect of particle size and particle generation rate on solid particle recovery efficiency was further investigated by analyzing the flow field distribution of the recovery unit. The results show that the maximum velocity of the gas phase in the recovery device increases with the increase of the absolute value of the outlet pressure, when the outlet pressure is -6500 Pa, the maximum velocity is 67.59 m/s. When the absolute value of the outlet pressure is greater than 6000 Pa, a small amount of steel shot particles is discharged from the recovery bin under the action of the outlet pressure, resulting in the loss of steel shot particles. After the outlet pipe position optimization, the steel shot particle recovery efficiency increased by 10% and the waste particle recovery efficiency increased by 18.9%.

## Introduction

Shot blasting treatment integrates surface peening, rust removal and cleaning, and is considered to be a highly efficient mechanical equipment. Because of its high processing efficiency, wide processing range and flexibility, it is widely used for cleaning and maintenance of mechanical parts, road and bridge and ship hull shells, and shot blasting machine is the equipment to realize shot blasting treatment^1^. Mobile shot blasting machine broke the traditional fixed shot blasting machine can only be cleaned indoors traditional parts of the limitations of the shot blasting process to expand to the surface of the ship rust removal and strengthening^2^, airport runway debonding repair, highway and bridge paint removal maintenance and various other fields, compared with other maintenance equipment, shot blasting treatment in the economy, environmental protection and efficiency has huge advantages^3^. Mobile shot blasting machine recycling device can realize the recycling of steel shot and automatic recycling of processing waste, but in the actual production and processing, the recycling rate of waste generated after shot blasting process is low^4^, and the dust particles generated during the recycling process are easy to cause environmental pollution, construction personnel are prone to lung diseases caused by inhalation of large amounts of dust^5^. Studies have shown that a more efficient and environmentally friendly recycling efficiency is achieved when the waste recovery efficiency reaches 90%^6^. Therefore, the efficient recycling of waste materials has received a lot of attention from researchers^7–9^.

Solid particles flowing with the air stream inside the blast machine recovery unit causes the gas phase (air)^10^ and solid phase (solid particles)^11^ to form a gas–solid two-phase flow inside the pipe^12^. Coupled simulation calculations of computational fluid dynamics and discrete element methods have gradually become a common method for numerical simulation of multiphase flows^13^. This coupled simulation technique uses the discrete element method^14^ to describe the solid-phase motion and perform particle information statistics by tracking individual particles along the system and calculating the particle motion state; while the computational fluid dynamics method is usually used to simulate the flow field of a continuous fluid phase (gas or liquid)^15^. The first application of the combined CFD-DEM model was reported to be the solid particle plug flow in a horizontal tube^16^, and then many others have been devoted to the analysis and simulation of various aspects of fluidized beds using coupled CFD-DEM calculations^17^. In the coupled solution process, the flow field is treated as a continuous phase and the solid phase is treated as a discrete phase, and the corresponding solutions for the flow field and the particle phase are obtained by the coupled calculation of the two software^18–20^.

Many researchers have used software coupling to perform calculations to predict the phase flow motion in pipelines and to investigate the gas flow in pipeline components and valves. Zhe^21^ studied the gas–solid two-phase flow characteristics and the erosion characteristics of a gate valve using CFD-DEM coupled calculation method. The experimental results showed that the number of particles upstream of the gate increases with decreasing valve opening; the number of particles plays an important role in the erosion. Lin^22^ used CFD-DEM coupled calculation method to analyze the two-phase flow and erosion characteristics of a concave cavity with slope, and the experimental results showed that the slope can transfer the erosion to the rear of the cavity, which reduces the maximum erosion rate of the wall of the cavity to some extent. Lin^23^ used CFD-DEM coupled calculation method to test the horizontal acrylic pipe as a test pipe, and the test solid The material was spherical polyethylene particles, and the gas–solid two-phase flow characteristics in the pipe were investigated, and by comparing with the finless and uniform soft fin gas–solid two-phase flow, the experimental results showed that the uniform soft fin reduced the conveying velocity and power consumption of the gas–solid two-phase flow in the pipe. Xu^24^ used the coupled computational CFD-DEM method to investigate the gas–solid two-phase flow characteristics of the mixture after threshing in a wheat threshing device, and the experimental results showed Zhang^25^ studied the gas–solid two-phase flow in the drill pipe at different rotational speeds using the coupled CFD-DEM calculation method, and the experimental results showed that the rotational speed of the drill pipe increased with the increase of the distance influenced by the cyclonic and airflow tangential velocity. The above mentioned scholars used CFD-DEM coupling method to calculate and study the flow characteristics of gas–solid two-phase flow in different devices, and achieved good engineering results.

Regarding the research on solid particle characteristics, Su^26^ studied the particle distribution and motion characteristics of particle charges in a cyclonic friction charger using CFD-DEM simulation method, and verified the validity of CFD-DEM model by comparing with experimental results. Lin^23^ studied the transport of polyethylene particles in a horizontal pipe using coupled CFD-DEM calculation method. The results showed that additional pressure losses were generated in the horizontal pipe in the lower gas velocity range. Chen^27^ used a discrete element method, Lagrangian-Lagrangian model to study the flow of liquid in the cylinder at high Reynolds coefficients and obtained the effect of St number on the distribution of solid particles. Li^28^ combined the CFD model with ANSYS Fluent and MATLAB based A coupled ANSYS-Fluent-based framework was proposed, and the computational results showed that regulating the valve aeration rate could alleviate the problem of uneven solid distribution, demonstrating the versatility of the new method. Vivacqua^29^ used the discrete unit method (DEM) to simulate particle flow, model the effects of shear strain rate, particle shape, and cohesion on flow characteristics, and validate the unified rheological description of powder The possibility of the existence of a unified rheological description of powder flow was verified. The aforementioned authors demonstrated the good results of coupled calculations for the calculation of the motion of solid particles in fluids.

In general, computational fluid dynamics methods and computational fluid dynamics-discrete element methods^30^ are the main methods for simulating gas–solid two-phase flows in pipes and enclosed devices^31,32^. Their reliability has been verified by a large number of experiments. Computational fluid dynamics ignores the interactions between particles, while the computational fluid dynamics-discrete element method considers the interactions between particles^33^. Therefore, the computational hydrodynamics-discrete element method is more accurate than the computational hydrodynamics method^34^. However, the computational fluid dynamics-discrete element method is much more costly than the computational fluid dynamics calculation method due to the calculation of particle interactions^35^.

The study of the problem of controlling the outlet pressure of the recovery device by an adjustable fan in the shot blasting machine, which affects the quality of the shot blasting process, is well established^2,7,8^. However, there is a lack of research on waste recovery. The influence of outlet pressure on the recovery efficiency of steel shot particles and scrap particles during the negative pressure pneumatic conveying process of shot blasting machines cannot be ignored^4^. Despite the importance of outlet pressure, the effect of outlet pressure particle recovery efficiency has not been studied to a large extent in the actual negative pressure sampling in enterprises^14^. Using computational fluid dynamics and discrete element method (CFD-DEM), the gas phase distribution of steel shot and waste particles within the recovery unit and the motion displacement characteristics of the particles during negative pressure recovery of the shot blasting machine at different outlet pressures were investigated. The effects of outlet pressure on the gas flow field, particle flow state and waste recovery efficiency during the negative pressure recovery process were further analyzed, and finally the location of the outlet pipe was optimized for the particle recovery efficiency, and these results can provide theoretical support for the design and operation of the shot blasting machine recovery system.

## Mathematical description

### Gas phase control equation

Based on the fact that the air in the shot blasting machine recovery unit is the carrier for transporting solid particles, the air is considered as incompressible and continuous phase, so the Navier–Stokes equation can be used to solve the gas-phase flow. Considering the small effect of temperature on the gas–solid two-phase flow and transport piping in the blast machine recovery unit, the effect of temperature in the solid particle recovery process is not considered. In numerical simulation, in order to make the equations closed and the simulation results closer to the actual situation, a suitable turbulence model should be selected. The standard *k–ω* model is based on the Wilcox *k–ω* model, which was modified to account for compressibility and shear flow propagation. The Wilcox *k–ω* model predicts the propagation rate of free shear flow, such as wake flow, mixed flow plate flow around, cylindrical flow around and radial jet flow. Therefore, it can be applied to wall bound flow and free shear flow. Considering the resistance of fluid to particles, gravity of particles, contact force between particles, baffle barrier and free shear flow, the Wilcox k-ω model can be applied to wall bound flow and free shear flow. So the Standard-*k–ω* model is used for calculation. The flow of the gas phase is consistent with the conservation of air mass and momentum, then the mass and momentum equations of the gas phase in the corresponding blast machine recovery unit are:

1 $$\frac{{\partial \rho_{{\text{a}}} }}{\partial t} + \nabla \cdot \left( {\rho_{{\text{a}}} v_{{\text{a}}} } \right) = 0$$

2 $$\frac{{\partial \left( {\rho_{{\text{a}}} v_{{\text{a}}} } \right)}}{\partial t} + \nabla \cdot \left( {\rho_{{\text{a}}} v_{{\text{a}}}^{2} } \right) = - \nabla p_{{\text{a}}} + \nabla \tau_{{\text{a}}} + \rho_{{\text{a}}} g - f_{{\text{a}}}$$

where *ρ*_a_ is the air density, kg/m^3^; t is the time, s; *v*_a_ is the air speed, m/s; *p*_a_ is the air pressure, Pa; g is the acceleration of gravity, m/s ^2^; *τ*_a_ is the air stress tensor, Pa; *f*_a_ is the average resistance of air, N.

A standard k-ε turbulence model is used to describe the rotating airflow in the recovery bin of a horizontal mobile blast machine, which is represented in the model as follows:

3 $$\frac{\partial }{\partial t}(\rho_{g} k_{g} ) + \frac{\partial }{{\partial x_{i} }}\left( {\rho_{g} k_{g} u_{i} } \right) = \frac{\partial }{{\partial x_{j} }}\left[ {\left( {\mu + \frac{{\mu_{t} }}{{\sigma_{k} }}} \right)\frac{\partial k}{{\partial x_{j} }}} \right] + G_{k} + G_{b} - \rho_{g} \varepsilon - Y_{M}$$

4 $$\frac{\partial }{\partial t}\left( {\rho_{g} \varepsilon } \right) + \frac{\partial }{{\partial x_{i} }}\left( {\rho_{g} \varepsilon u_{i} } \right) = \frac{\partial }{{\partial x_{j} }}\left[ {\left( {\mu + \frac{{\mu_{t} }}{{\sigma_{\varepsilon } }}} \right)\frac{\partial \varepsilon }{{\partial x_{j} }}} \right] + C_{1\varepsilon } \frac{\varepsilon }{{k_{g} }}\left( {G_{k} + C_{3\varepsilon } G_{b} } \right) - C_{2\varepsilon } \rho_{g} \frac{{\varepsilon^{2} }}{{k_{g} }}$$

where, *k*_*g*_ is the turbulent kinetic energy, *ε* is the turbulent dissipation rate, $\mu$ is the dynamic viscosity of the gas, and the model constants *C*_1*ε*_、 *C*_2*ε*_、C3ε,、$\sigma_{k}$ and $\sigma_{\varepsilon }$ have the following default values: *C*_1*ε*_ = 1.44, *C*_2*ε*_ = 1.92, *C*_3*ε*_ = 1.3, $\sigma_{k}$ = 1.0, $\sigma_{\varepsilon }$ = 1.3. $\mu_{t}$ is the turbulent viscosity, which is expressed as follows

5 $$\mu_{t} = \rho_{g} C_{\mu } \frac{{k^{2} }}{\varepsilon },C_{\mu } = 0.09$$

### Solid phase control equation

Given that the air in the blast machine recovery unit carries the solid particle motion, the solid particle population is considered as a discrete phase, so the flow of solid particles can be solved by DPM. Based on the Lagrange coordinate system^37^, the trajectory of the discrete phase is solved by the differential equation of particle forces to simulate the particle motion in turbulent flow. Then the expression of the differential equation of particle force in the discrete phase is:

6 $$\left\{ {\begin{array}{*{20}l} {\frac{{{\text{d}}v_{{\text{p}}} }}{{{\text{d}}t}} = F_{{\text{d}}} \left( {v_{{\text{a}}} - v_{{\text{p}}} } \right) + \frac{{g\left( {\rho_{{\text{p}}} - \rho_{{\text{a}}} } \right)}}{{\rho_{{\text{p}}} }} + F_{{\text{other }}} } \hfill \\ {F_{{\text{d}}} = \frac{18\mu }{{\rho_{{\text{p}}} d_{{\text{p}}}^{2} }}\frac{{C_{{\text{d}}} Re_{{\text{p}}} }}{24}} \hfill \\ {Re_{{\text{p}}} = \frac{{\rho_{{\text{a}}} d_{{\text{p}}} \left| {v_{{\text{p}}} - v_{{\text{a}}} } \right|}}{\mu }} \hfill \\ {C_{{\text{d}}} = a_{1} + \frac{{a_{2} }}{{Re_{{\text{p}}} }} + \frac{{a_{3} }}{{Re_{{\text{p}}}^{2} }}} \hfill \\ \end{array} } \right.$$

where *v*_p_ is the particle velocity, m/s; *F*_d_(*v*_a_-*v*_p_)is the traction force per unit mass of particles, m/s^2^; *ρ*_p_ is the particle density, kg/m^3^; *μ*is the aerodynamic viscosity, Pa·s; *C*_d_is the traction coefficient; *Re*_p_ is the relative Reynolds number of particles; *d*_p_ is the particle size, m; *F*_other_is the other force per unit mass of particles, m/s^2^; *a*_1_、*a*_2_、*a*_3_ are constants.

Considering the actual working conditions, the two-way coupling method of CFD-DEM is used to simulate the transport process of gas and solid phases in the shot blasting machine recovery unit^38^. Based on the above controlling equations for the gas and discrete phases, the momentum value transmitted from the gas phase to the discrete phase is solved by calculating the momentum change of the particles when they pass through a defined spatial region in the flow field, thus realizing the two-way coupling calculation^39,40^.

**Figure 1:**
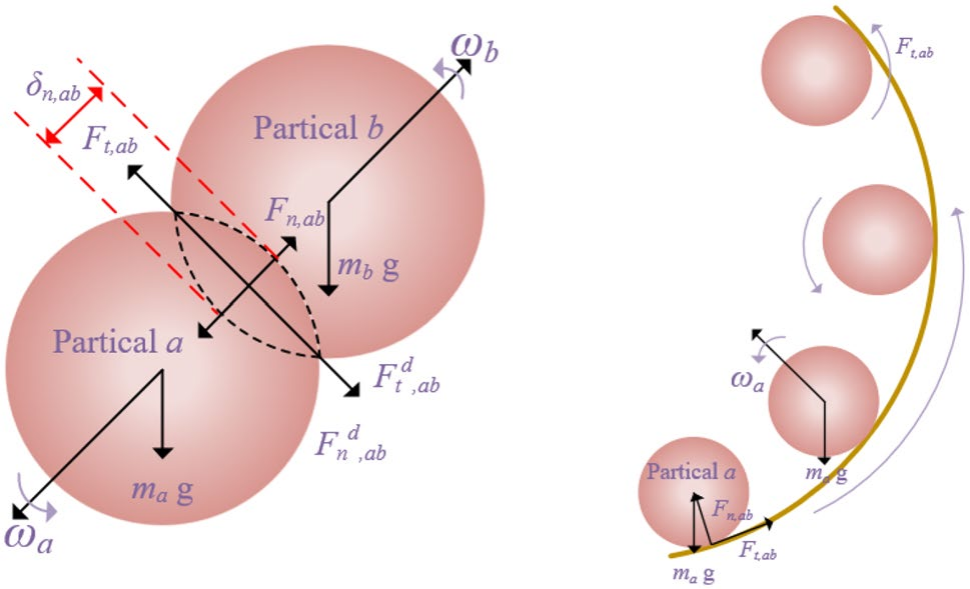
Schematic diagram of particle collision.

The contact forces between particles *a*,*b* and the pipe wall on particle *a*, are shown in Fig. 1. The above rotational motion of particle *a* is described as:

7 $$I_{a} \frac{{d\omega_{a} }}{{d_{t} }} = \sum\limits_{b = 1}^{{k_{a} }} {(F_{c,ab} + F_{d,ab} )}$$

where, $I_{a}$ and $\omega_{a}$ denote the moment of inertia tensor and rotational velocity of particle a, respectively. $F_{c,ab}$ is the tangential force acting on particle a by particle b. $F_{c,ab}$ is defined as follows:

8 $$F_{c,ab} = R_{a,b} \left( {F_{n,ab} + F_{t,ab} } \right)$$

9 $$F_{n,ab} = \frac{4}{3}E^{ * } \sqrt {R^{ * } } \delta_{n,ab}^{1.5}$$

10 $$F_{t,ab} = - 2\sqrt{\frac{5}{6}} \frac{\ln e}{{\sqrt {\ln^{2} e + \pi^{2} } }}\sqrt {S_{n} m^{*} } v_{n,ab}$$

11 $$S_{n} = 2E^{ * } \sqrt {R^{ * } \delta_{n,ab} }$$

where,$F_{n,ab}$ is the normal contact force, $F_{t,ab}$ is the tangential contact force, $R_{a,b}$ is the vector from the center of mass to the point of contact, $S_{n}$ is the normal stiffness, $\delta_{n,ab}^{{}}$ is the normal overlap, $E^{ * }$,$R^{ * }$, $m^{*}$ $v_{n,ab}$ and $e$ is the equivalent Young's modulus, equivalent radius of the particle, equivalent mass, relative normal velocity and recovery factor.

$F_{d,ab}$ is the rolling friction moment of particle b acting on particle a, $F_{d,ab}$ defined as follows:

12 $$F_{d,ab} = - \mu_{r,ab} d_{a} \frac{{\omega_{ab} }}{{\left| {\omega_{ab} } \right|}}$$

where, $\mu_{r,ab}$ and $d_{a}$ are the rolling coefficient and particle diameter, respectively, $\omega_{ab}$ and is the relative angular velocity of particle a to particle b. During the particle–particle collision, the forces and moments on the particle are similar to those in the particle–particle collision, that is, Eqs. (8)–(12).

## Model settings

### Geometric models

**Figure 2:**
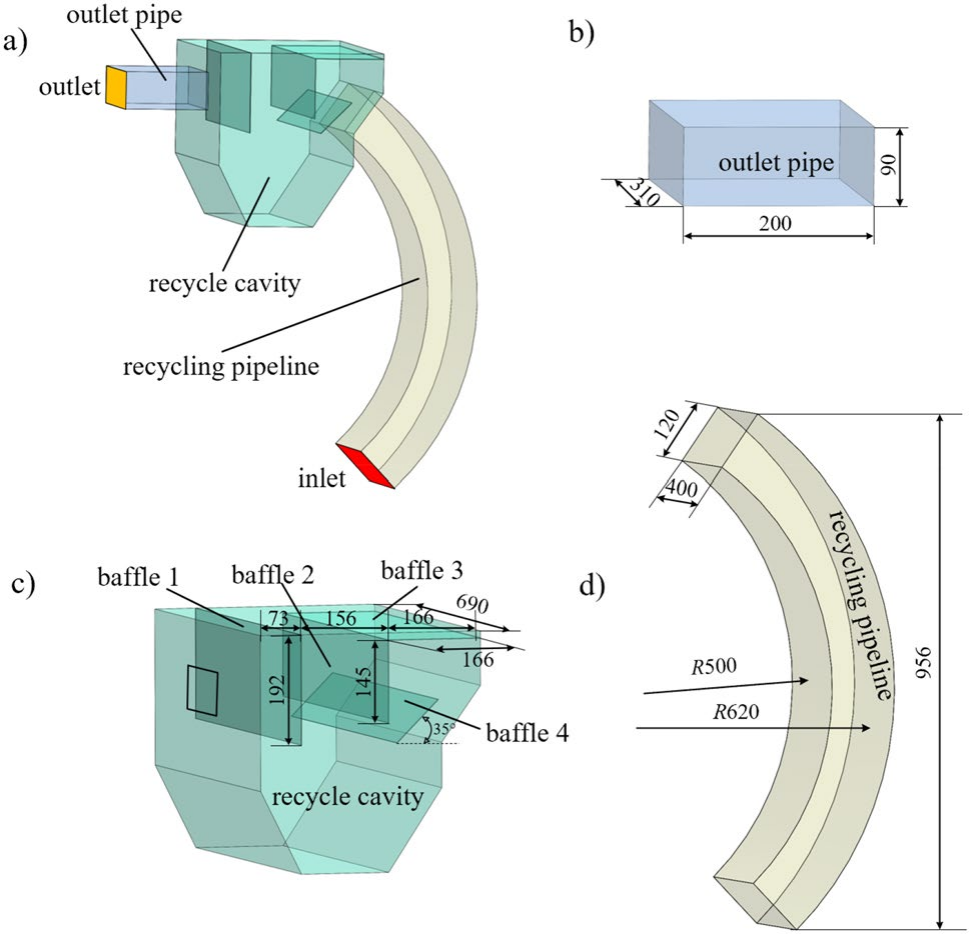
Shot blasting machine recovery device and dimensional drawing: (**a**) overall view of recovery unit; (**b**) outlet pipe; (**c**) recovery bin; (**d**) recovery pipe line.

The structure of the shot peening recovery device studied in this paper is shown in Fig. 2a), the size of the outlet pipe is shown in Fig. 2b), and the structure of the recovery bin and the size of the recovery pipe are shown in Fig. 2c,d, which show the relative position of the simplified recovery bin and its internal baffle used in this study. In order to make the model close to the actual working condition, besides ensuring the accuracy of the relative position of the baffle, In addition, the characteristics of gas–solid two-phase flow and the movement of particles are considered in parameter setting.

The finite element software FLUENT and EDEM were used to perform numerical simulations. In this study, involving both gas phase (air) and solid phase (steel shot particles and waste particles), the Euler–Lagrange method^41^ was used to solve the flow field, the Navier–Stokes equations^42^ for incompressible viscous fluids were solved in the Eulerian coordinate system and the particle equations of motion were solved in the Lagrangian coordinate system.

During the shot blasting process, the steel shot and its scrap generated after striking the surface of the part are mixed into one, forming a particle population with the coexistence of two kinds of particles, and the particle population flows with the airflow, forming a gas–solid two-phase flow under the effect of fluidization^43^, and the phase flow flows in the recovery pipe and enters the recovery bin, and the baffle in the recovery bin constrains the flow of gas, and the steel shot particles fall into the In this test and simulation, it is considered that all waste particles excluded from the outlet of the recovery bin can be recovered by the dust collector.。

### Simulation conditions

#### Grid independence verification

**Figure 3:**
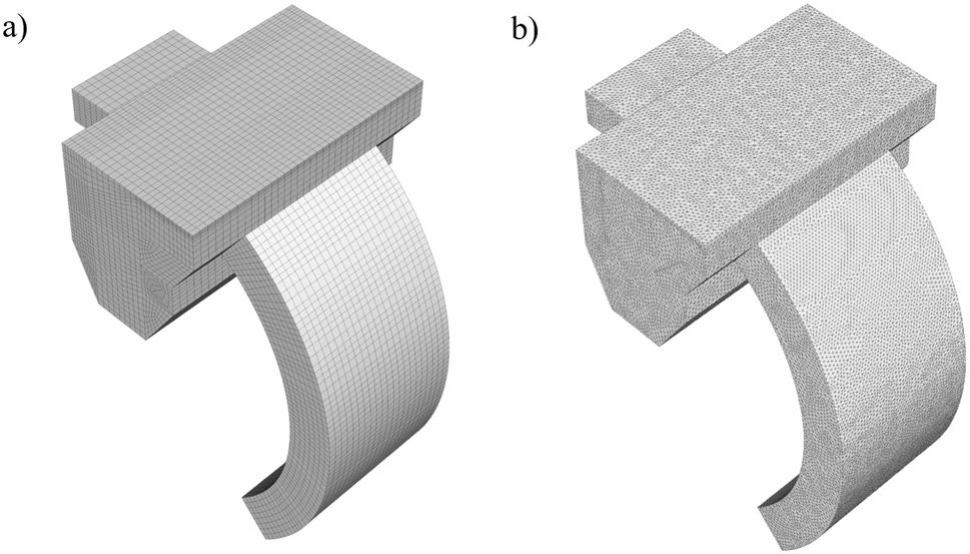
Grid diagram of the recovery unit: (**a**) structural grid; (**b**) non-structural grid.

The ICEM-CFD slab in ANSYS finite element simulation software was used for the mesh delineation of the shot blasting machine recovery device, and considering the calculation speed and the quality of the mesh generation, the structural type mesh^44^ was used for the delineation by comparing the characteristics of structural and non-structural meshes, and the resulting mesh is shown in Fig. 3.

**Figure 4:**
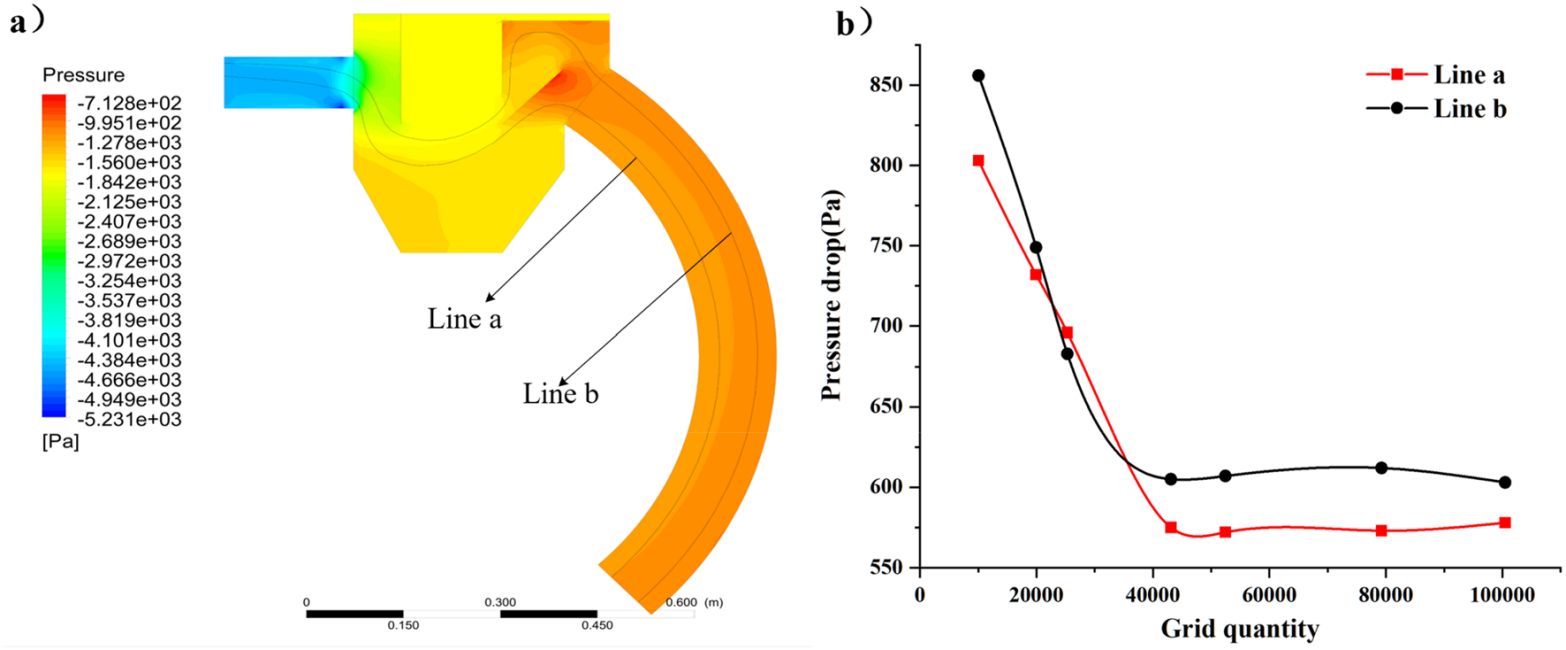
Mesh irrelevance analysis: (**a**) measuring position (**b**) pressure drop varies with the number of grids.

As shown in Fig. 3, the recycling bin model was meshed and the entrances and exits were locally encrypted. Grid independence tests were performed on the numerical model. The number of these seven grids are 10,058, 19,934, 25,293, 43,083, 52,432, 79,256 and 100,458 describes the pressure difference between the outlet and inlet after 2500 iterations of fluid reaching steady state at velocity inlet and pressure outlet. As shown in Fig. 4, when the number of grids increases from 43,083 to 52,432, the pressure loss value of the whole device changes slightly, and at this time it is considered that the grids of 43,083 and 52,432 reach grid independence, so the grid of 43,083 is chosen for the simulation, which can not only reduce the calculation volume, but also obtain a higher calculation.

#### Particle characterization

**Figure 5:**
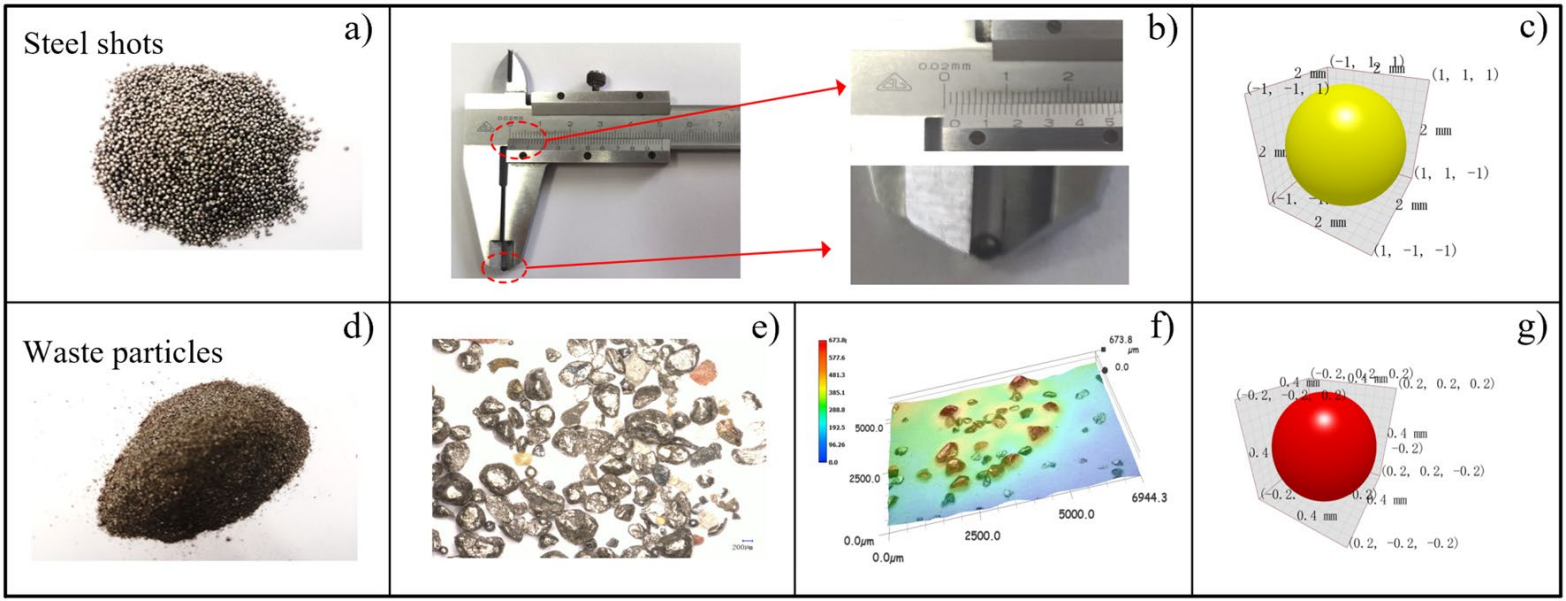
Simplified process diagram of particles (**a**) steel shot physical picture (**b**) steel shot size measurement (**c**) simplified steel shots model (**d**) physical diagram of waste (**e**) particle magnification diagram (**f**) waste particles under ultra-depth of field microscope (**g**) simplified waste particles model.

The steel shot particles and scrap particles were obtained through field sampling, and the main composition of the scrap particles was iron oxide particles and debris after testing, as shown in Fig. 5a,d; then the observation of the scrap particle size was carried out using an ultra-deep field microscope, and the distribution of the particle diameter size was 0.3–0.5 mm, and the average value of 0.4 mm was taken as the scrap particle diameter, which can better express the scrap The size characteristics of the particles, as shown in Fig. 5e,f; using vernier calipers to measure the size of the steel shot particle size, the diameter size of 2 mm, as shown in Fig. 5b.

#### Analog parameter setting

The Hertz-Mindlin contact model was used to explain particle–particle, particle–wall, and particle-baffle contacts in performing particle-phase calculations^45^. The coupling calculations were performed using the Euler–Lagrange model and gravity, lift (Magnus and Besset), inertia forces, friction and contact forces were considered during the motion of the particles. The CFD and DEM time steps were set to 1e−4 s and 2.5e−7 s, considering the size of the solid particles and the reasonableness of the software coupling calculation time^43^. In this case, the CFD time step was set to 400 times the DEM time step^47^. The wall material properties of the steel shot particles, scrap particles, and the shot blast machine recovery bin are shown in Table 1.

**Table 1:** EDEM simulation parameter settings.

Name	Type	Numerical value
Steel pellets	Poisson's ratio [–]	0.25
	Density [kg/m^3^]	7.2e+3
	Shear modulus [Pa]	7.3e+10
Scrap pellets	Poisson's ratio [–]	0.25
	Density [kg/m^3^]	5240
	Shear modulus [Pa]	2e+9
Wall	Poisson's ratio [–]	0.3
	Density [kg/m^3^]	7.85e+3
	Shear modulus [Pa]	7.3e+10
Pellets-Pellets	Elastic recovery factor [–]	0.25
	Static friction coefficient [–]	0.75
	Rolling friction coefficient [–]	0.05
Particle–wall	Elastic recovery factor [–]	0.5
	Static friction coefficient [–]	0.5
	Rolling friction coefficient [–]	0.05

According to the actual working conditions of the negative pressure pneumatic conveying of the shot blasting machine, the outlet and inlet pressures are obtained and parameterized, setting the inlet as pressure inlet and the outlet as pressure outlet. Set two particle factories for steel shot and scrap generation, respectively, with 2000 particles per second generation capacity of particles respectively^45^. In fact, the diameter of scrap particles varies from microns to millimeters during the sampling process of the enterprise processing. The parameters of the inlet and outlet in Fluent software are shown in Table 2.

**Table 2:** FLUENT simulation parameter settings.

Name	Type	Numerical value
Entrance	Type [–]	Pressure inlet
	Hydraulic diameter [mm]	139.5
	Turbulence density [–]	5%
Export	Type [–]	Pressure outlet
	Hydraulic diameter [mm]	184.62
	Turbulence density [–]	5%
Air	Density [kg/m^3^]	1.225
	Viscosity [kg/m^−1^]	7.85e+3
	Specific heat capacity [j/kg^−k^]	1006.43

In order to improve the recovery efficiency of waste particles, reduce waste particles mixed with steel shot and air pollution, companies often use to increase the speed of the fan, reduce the pressure value at the exit of the shot blasting machine recovery device to carry out further recovery of waste particles, but companies often use the empirical method of export pressure settings, the lack of particle movement and airflow distribution of theoretical support, failure to systematically elaborate the shot blasting machine recovery bin exit pressure and the objective law of waste particle recovery efficiency. For this reason, the negative outlet pressure of − 4000, − 4500, − 5000, − 5500, − 6000 and − 6500 Pa is used for simulation tests, and in the analysis of the results in section "Results and discussion", the absolute value of the pressure is used for the analysis of the outlet pressure in order to make the analysis results more simple and clear, and to explore the relationship between the outlet pressure and the steel shot particles and scrap recovery efficiency.

**Figure 6:**
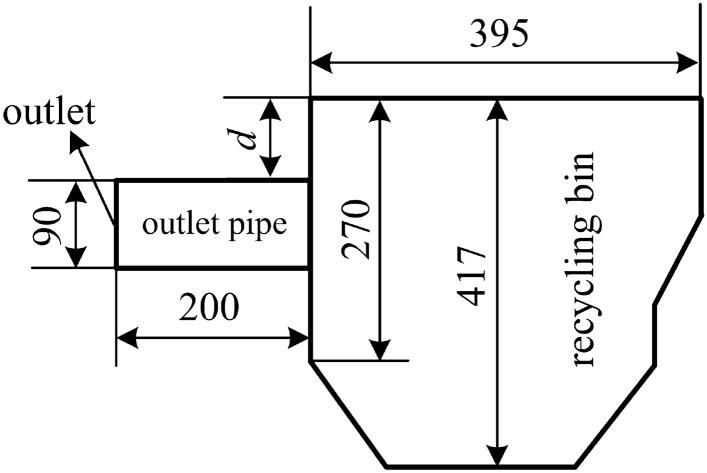
Setting of outlet pipe position (*d*).

Different outlet locations have a large impact on the airflow distribution in the recovery bin, and it is important to study the effect of different locations of the outlet duct on the waste particles, i.e., the size of d as shown in Fig. 6: 0 mm, 30 mm, 60 mm, 90 mm, 120 mm, 150 mm and 180 mm (as shown in Fig. 6).

However, this paper initially investigated the effect of the magnitude of the outlet pressure and the relative position of the outlet pipe of the recycling bin of the shot blasting machine under negative pressure conditions on the negative pressure recovery efficiency of the scrap, where the particle size of the scrap particles was larger than the conventional sampling method^48^. The effects of waste particles, shot diameter and the relative position of the internal baffle of the recovery bin on the waste recovery efficiency will be explored in the future.

## Results and discussion

The inhomogeneity of the distribution of waste material particles within the blast machine recovery unit is an inherent characteristic of gas–solid two-phase flow, and particle distribution is influenced by gas velocity, particle mass flow rate, and material shape. Optimization and scale-up of the blast machine recovery unit is not yet fully understood as further research is needed on measurement devices and methods for particle identification and tracking. In this section, the flow behavior of waste particles and gas phase is revealed by CFD-DEM method and the effect of outlet pressure on particle motion behavior is investigated.

### Gas phase velocity distribution and model validation

#### Gas phase flow characteristics

**Figure 7:**
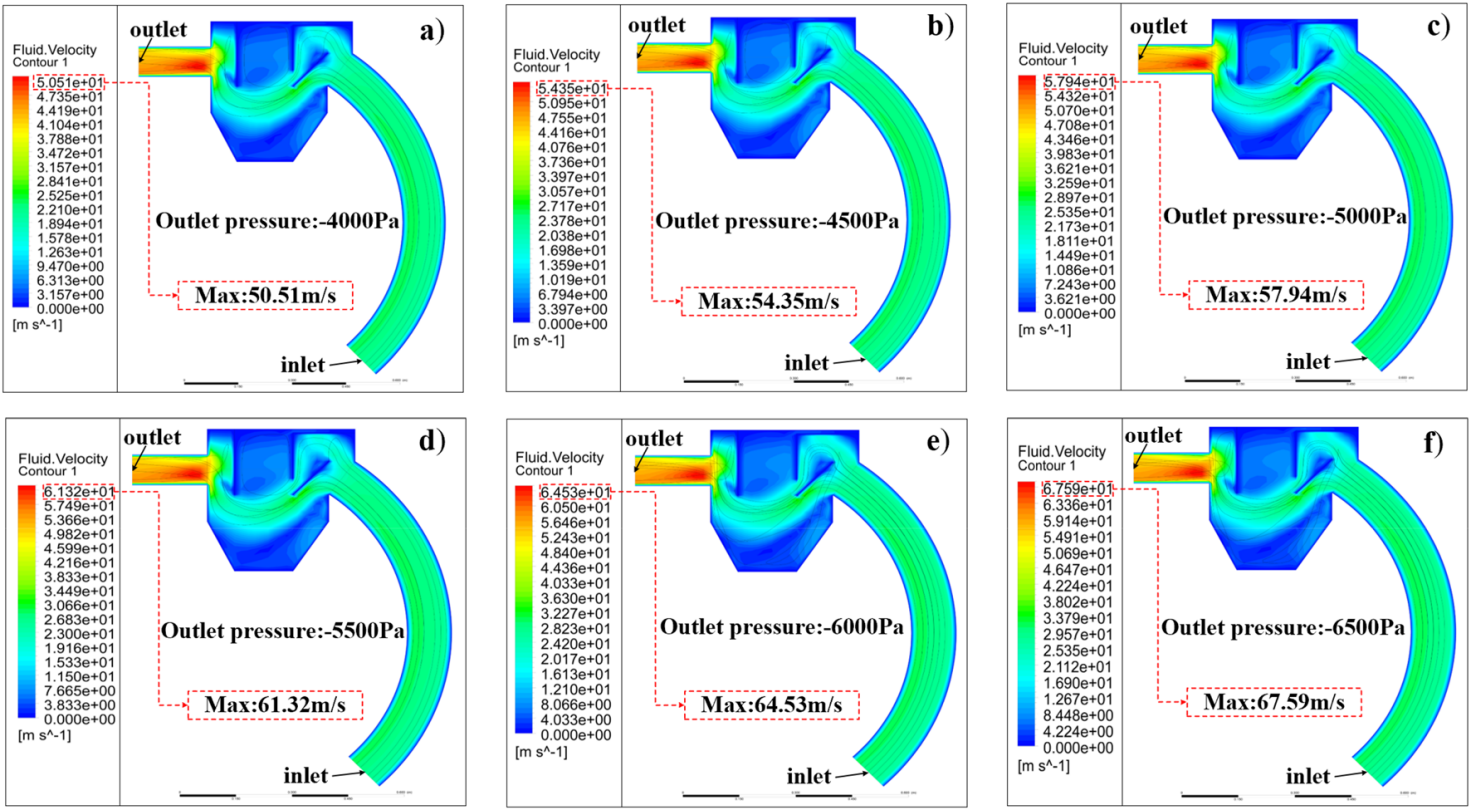
Airflow distribution and flow lines in the shot blasting machine recovery unit.

The particle recovery system within the shot blasting machine recovery unit provides a large space for the separation and recovery of steel shot particles and waste particles. Considering the complexity of the structural distribution inside the device, the interaction force between the gas phase and the solid phase is large, the collision of solid particles is violent, and the fluid dynamics is complex. Figure 7 shows the characteristics of the fluid transient velocity distribution from the inlet to the outlet of the recovery unit and the form of the flow distribution inside the unit. The flow characteristics of the two particle mixtures vary with the decrease of the pressure value at the outlet of the device from − 4000 to − 6500 Pa, as shown by the increase of the maximum value of the fluid velocity from 50.51 to 67.59 m/s, which leads to different transport efficiency and degree of separation.

Figure 7 shows the core-step structure of the steel shot, waste particles and fluid in the blast machine recovery unit. The wind velocity is greatest and unevenly distributed on the right side of the exit duct. The cross-sectional area of the shot blast recovery unit is not constant; therefore, the velocity and instantaneous velocity direction of the gas phase varies with particle transport time. The velocity magnitude within the recovery duct was uniformly distributed. In addition, the generation of two vortices was observed in the recovery bin, where the high velocity and low pressure caused the pressure loss in the recovery unit.

Due to the existence of particle phase in the recovery pipe, particles collide and move on the outside of the recovery pipe under the action of conveying force, inertia force, gravity and lift force of airflow, when the particle speed is less than the air speed, the particles make accelerated motion; when the particle speed is greater than the air speed, the particles make decelerated motion. Due to the special internal structure of the recovery pipe, the airflow on the inner side is more likely to be blocked by the inner wall of the recovery pipe when moving, resulting in the inner airflow speed being smaller than the outer airflow speed. This phenomenon has been present during the process of outlet pressure reduction in the shot blasting machine recovery unit steel shot and scrap recovery process.

As shown in Fig. 7a–f, the blast machine recovery device in the recovery process, with the outlet pressure decreases, the gas phase velocity distribution in the device changes less, and the maximum velocity is proportional to the absolute value of the negative outlet pressure value, which is in line with the law of gas phase velocity changes measured by companies in the actual process.

The streamline describes the flow path of the air in the blast machine recovery device and is able to represent the velocity direction of the different fluid masses at that moment, as can be derived from the streamline diagrams in Fig. 7a–f, when the outlet pressure is − 4000 to − 5500 Pa, the streamline enters the recovery bin along the recovery pipe, and under the blocking effect of the baffle 4, the gas is divided into two jets, one passing from above the baffle 4 and the other One part passes from the lower side of baffle 4. The jet passing from the upper side of baffle 4 enters the area enclosed by it and baffle 1, and after flowing for some distance in the area, the velocity reaches to get rid of the adsorption force in the area due to the velocity gradient. After passing the lower side of baffle 4, the two jets merge together into a high-speed jet that flows toward the exit pipe.

When the outlet pressure is − 5500 to − 6500 Pa, the airflow speed inside the whole device increases and the pressure decreases, the airflow passing from the lower side of the baffle 4 flows toward the bottom of the recovery bin under the effect of the pressure difference and the special internal structure of the recovery bin, after a period of time, the airflow finally flows toward the outlet under the effect of the negative pressure at the outlet and flows out of the device by the outlet.

#### Shot blast machine recovery model accuracy verification

**Figure 8:**
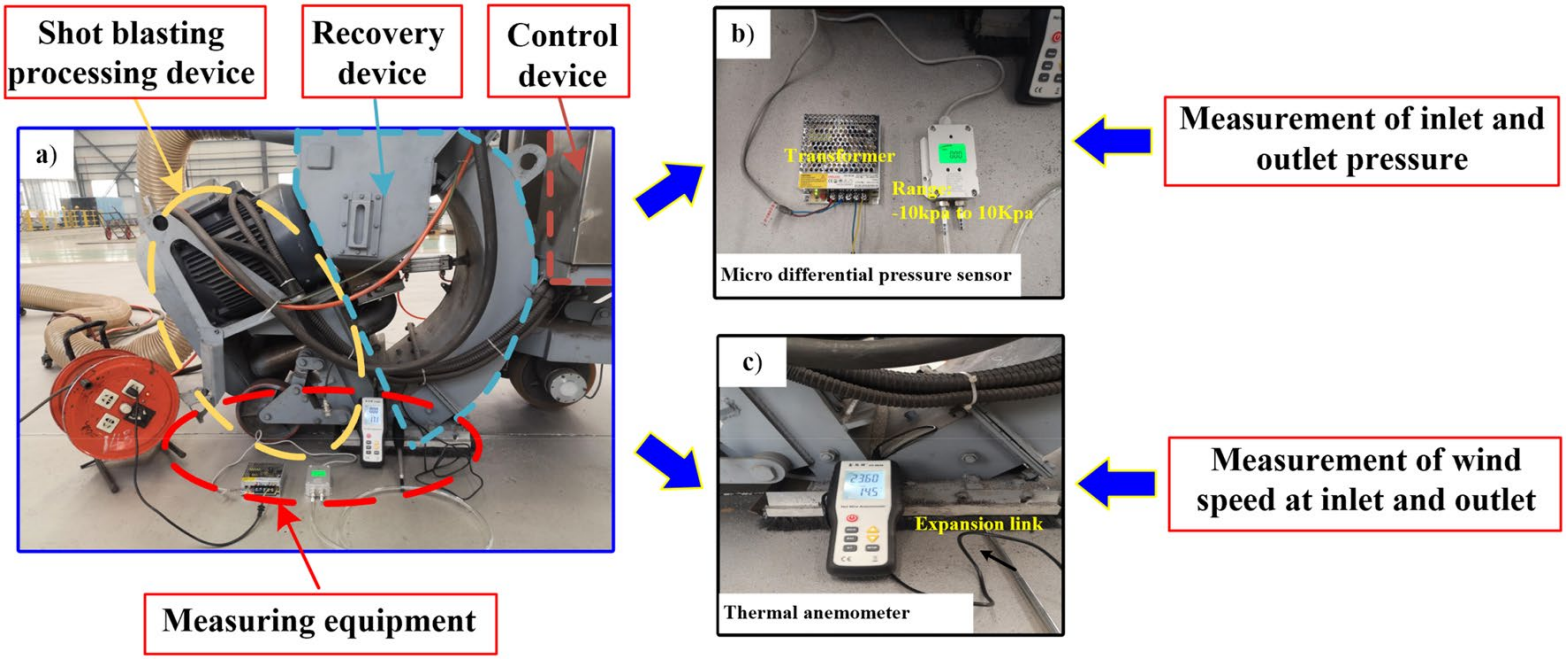
Model accuracy verification test equipment.

In order to verify the accuracy of the calculation of the proposed simulation model, a comparison between the test results and simulation results is used to verify. As shown in Fig. 8, the wind speed at different locations of the outlet and inlet of the shot blasting machine recycling device is measured by a thermal anemometer, and the pressure value is measured by a micro differential pressure sensor.

Table 3 shows the data measurement position, taking the outlet pressure value of − 4000 Pa as an example, the inlet cross-section size is a rectangle of 400 mm × 120 mm, and after taking 2 × 5 points at equal distance, the thermal anemometer working port is put into the corresponding point, and then the wind speed is measured and recorded there. The outlet cross-section size is 310 mm × 90 mm rectangle, and the measurement points are selected in the same way as the inlet, and the wind speed magnitude at each point is recorded. The simulation results calculated by the established recovery device model are shown in Table 3. The same position as the test measured out and inlet was used to extract the velocity at the corresponding position, and the wind speed at the corresponding position was recorded as the result of the simulation calculation.

**Table 3:**
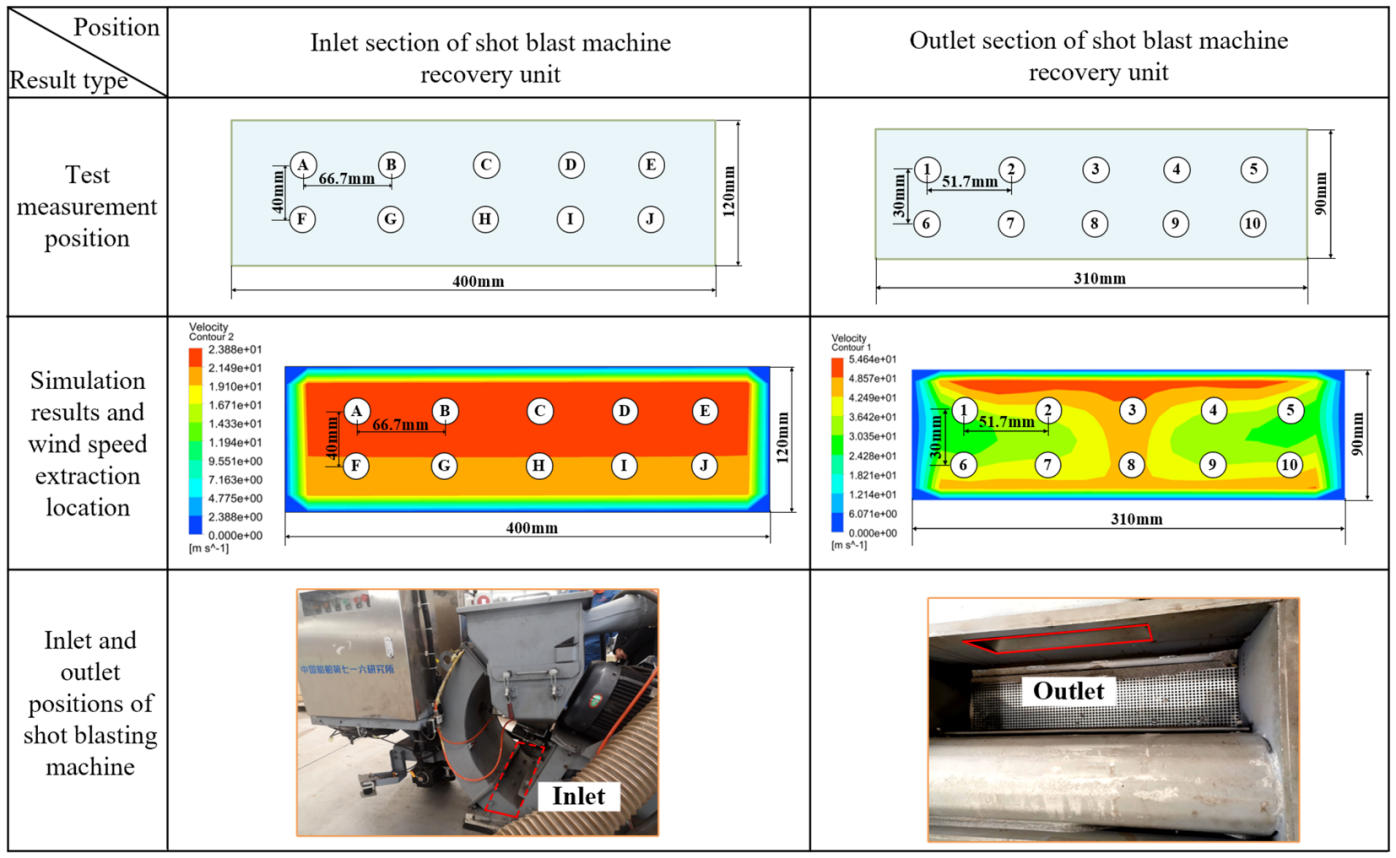
Data measurement locations.

**Figure 9:**
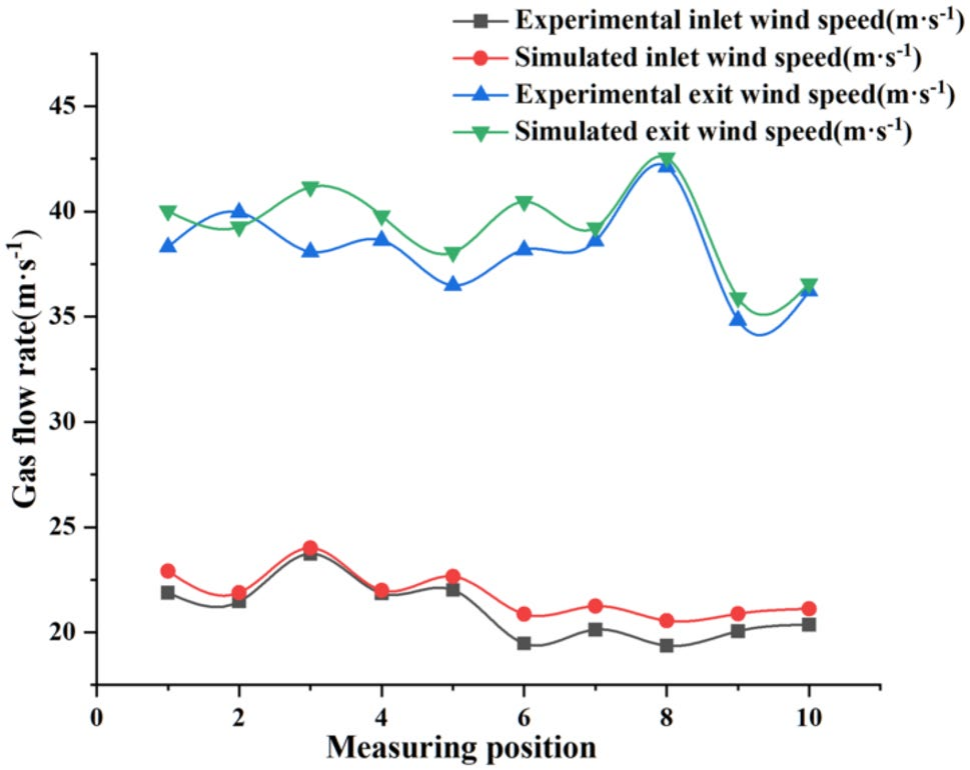
Comparison of gas phase test and simulation results.

The gas phase velocities in the simulated data were compared with the experimental data. As shown in Fig. 9, it can be concluded from the comparison results that the experimental parameters are in good agreement with the simulated parameters. This proves that the shot blast machine recovery model is accurate and can be used for subsequent simulation calculations.

### Gas–solid two-phase flow characteristics

#### Steel pellet trajectory

**Figure 10:**
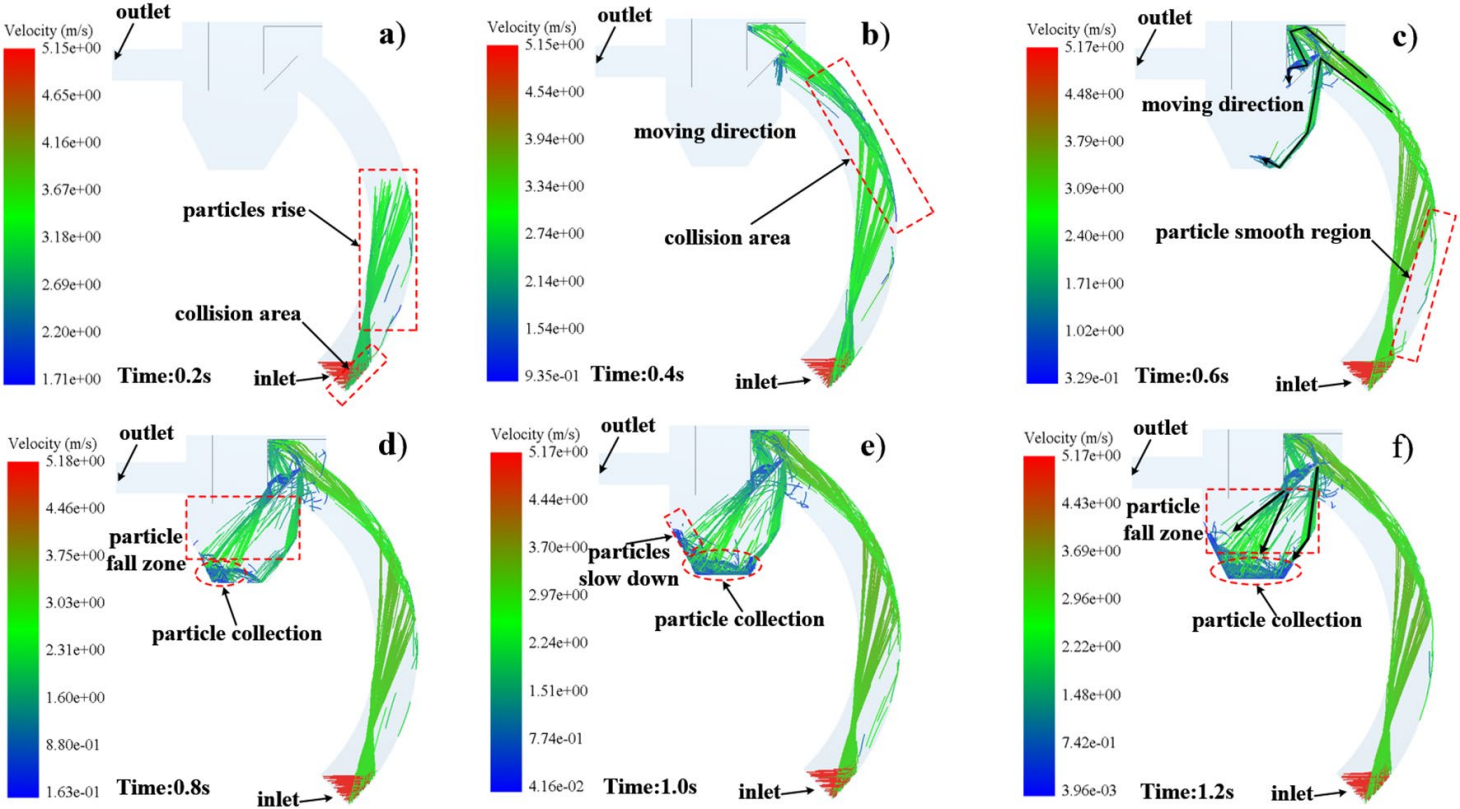
Steel shot particle motion characteristics.

In gas–solid two-phase flow motion, in addition to the analysis of the gas phase distribution and gas flow characteristics within the recovery unit, the properties and motion characteristics of the solid phase—particles are also necessary. The trajectory of solid particles is the core parameter for the operation of the blast machine recovery unit and solid particle recovery. Figure 10 shows the motion trajectory of the particles inside the blast machine recovery unit using a coupled CFD-DEM calculation method.

As shown in Fig. 10a, from the process of particle generation to 0.2 s, the particles enter the inlet of the recovery pipe along the preset angle, and the steel pellets move some distance under the action of initial velocity and airflow and then collide with the outer wall of the recovery pipe, because the wall is curved, the surface of the steel pellets colliding with the wall can be compared to a tiny plane, and the angle of incidence of the steel pellets is equal to the angle of reflection after collision. After the steel shot collision, along different angles, under the airflow transport upward movement, a small number of particles in the lowest part of the recovery pipe collision with the wall, at this time the collision angle is larger, less momentum loss, get rid of the airflow transport force, in the role of inertia along the wall sliding.

The movement of the steel shot within 0.2–0.4 s is shown in Fig. 10b. The rising steel shot touches the wall again, and after the collision the steel shot moves in the tangential direction of the recovery pipe and enters the recovery bin. Due to the existence of baffle 4, the steel shot is divided into two streams after entering the recovery bin. As can be seen from Fig. 10c, the particles passing under the baffle 4 are reflected at an angle of 85°–95° from the horizontal surface after impacting on the lower surface of the baffle, and the velocity increases from 1.02 to 3.09 m/s toward the bottom of the recovery bin under the action of gravity and inertia force.

The particles passing above the baffle 4, after hitting the baffle 3, baffle 2 and the upper side of the baffle 4 at one time the speed drops to 0.45 m/s and then along the inclination angle of the baffle 4, enter the particle landing zone under the action of gravity and inertia force and finally fall to the bottom of the recovery bin as shown in Fig. 10d. In the particle deceleration zone in Fig. 10e, the combined force of inertia force, lift force and airflow conveying force of the particles is less than the gravity of the steel shot itself, so it does deceleration movement, and then falls into the particle recovery zone under the action of gravity.

#### Waste material movement characteristics

**Figure 11:**
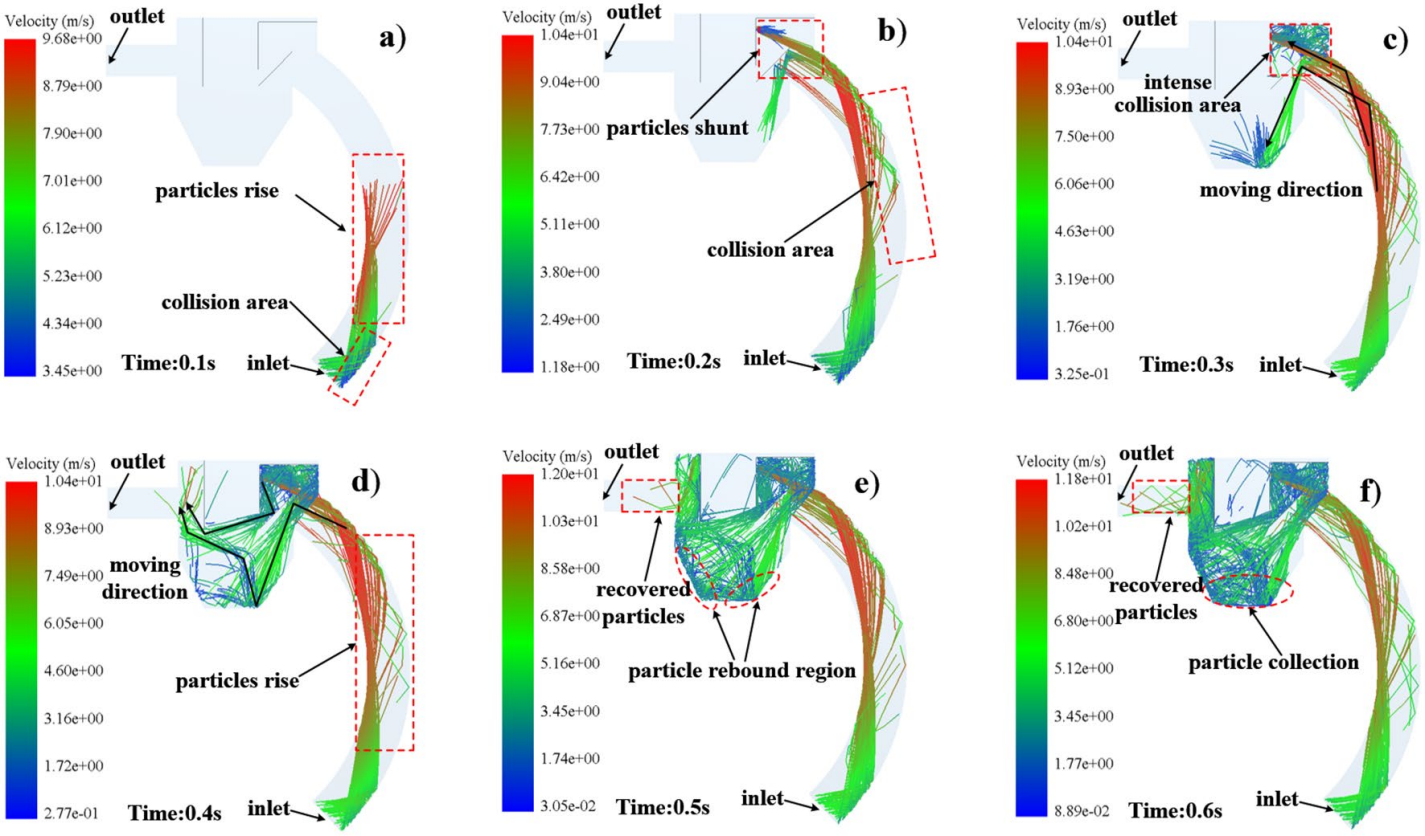
Velocity and trajectory of waste material particles movement.

The analysis of the trajectory and velocity magnitude of the waste material at any given moment is also necessary. The trajectory of the waste particles can directly reflect the movement state of the particles, and the movement characteristics of the waste particles are the core of the operation of the shot blasting machine recycling unit, solid particle recovery and environmental protection requirements (Fig. 11). The motion characteristics of the waste particles in the shot blasting machine recycling unit are calculated using the coupled CFD-DEM calculation method.

Different from the steel shot, the waste particles, due to their small particle size, are less affected by inertia, gravity and centrifugal force, and are more likely to change their original trajectory when they are subjected to the force of airflow, as shown in Fig. 11a, and the trajectory of the steel shot is different. After the collision with the outer wall of the pipe, the speed is reduced from 6.21 to 3.58 m/s, with serious loss of kinetic energy, and then accelerated upward under the conveying force of airflow after rebounding, at which time the maximum speed of the waste particles reaches 9.68 m/s.

The waste particles move up in the recovery pipe, and the particles that collide with the wall in the collision zone in Fig. 11b bounce back after colliding with the lower face of baffle 4, and the particles move toward the bottom of the recovery bin, and the particles that do not go through the collision zone enter the recovery bin from the upper end of baffle 4. From the intensive impact zone in Fig. 11c, the impact between the waste particles and baffle 2, baffle 3 and baffle 4 is more frequent, and the kinetic energy loss of the waste particles is the most serious at this time. When the waste particles are gathered, as shown in Fig. 11d, the direction of movement of the waste particles separated by baffle 4 is marked by the black arrows in the Fig. 11, and the final waste moves toward the direction of the exit.

According to the analysis of the gas phase flow characteristics in “Gas phase flow characteristics”, the gas phase distribution at the bottom of the recovery bin shows that a part of the waste particles passing under the baffle 4 cannot enter the outlet pipe under the air flow after entering the bottom of the recovery bin, but enter the inlet port with the steel shot under the action of vortex and particle impact, this part of the waste particles cannot be recovered, and this part of the particles will not only cause the key parts of the blast machine blade Wear (some data show^5^, when the content of waste particles in the shot every 1% increase, the wear of the blast machine blade increased by 2–3 times.) Also, during the shot peening process, the waste particles are discharged into the air through the shot peening process, causing particle dust pollution to the atmosphere. In Fig. 11e,f, the particles entering the exit duct can be smoothly discharged into the recovery bin and into a special dust recovery device for recovery.

#### Effect of outlet pressure on pellet recovery efficiency

According to the settings of the outlet pressure in 3.3.3, the outlet pressure was set and simulated, and the gas phase was calculated and the results analyzed by Fluent; the particle phase was calculated by EDEM software for the properties of particle velocity, trajectory and number. In order to explore the effect of the size of the outlet pressure on the particle recovery efficiency, the number of particles needs to be counted in the plate of Setup Selections-Grid Bin Group in the post-processing of EDEM discrete element software to further calculate the recovery efficiency of both particles.

**Figure 12:**
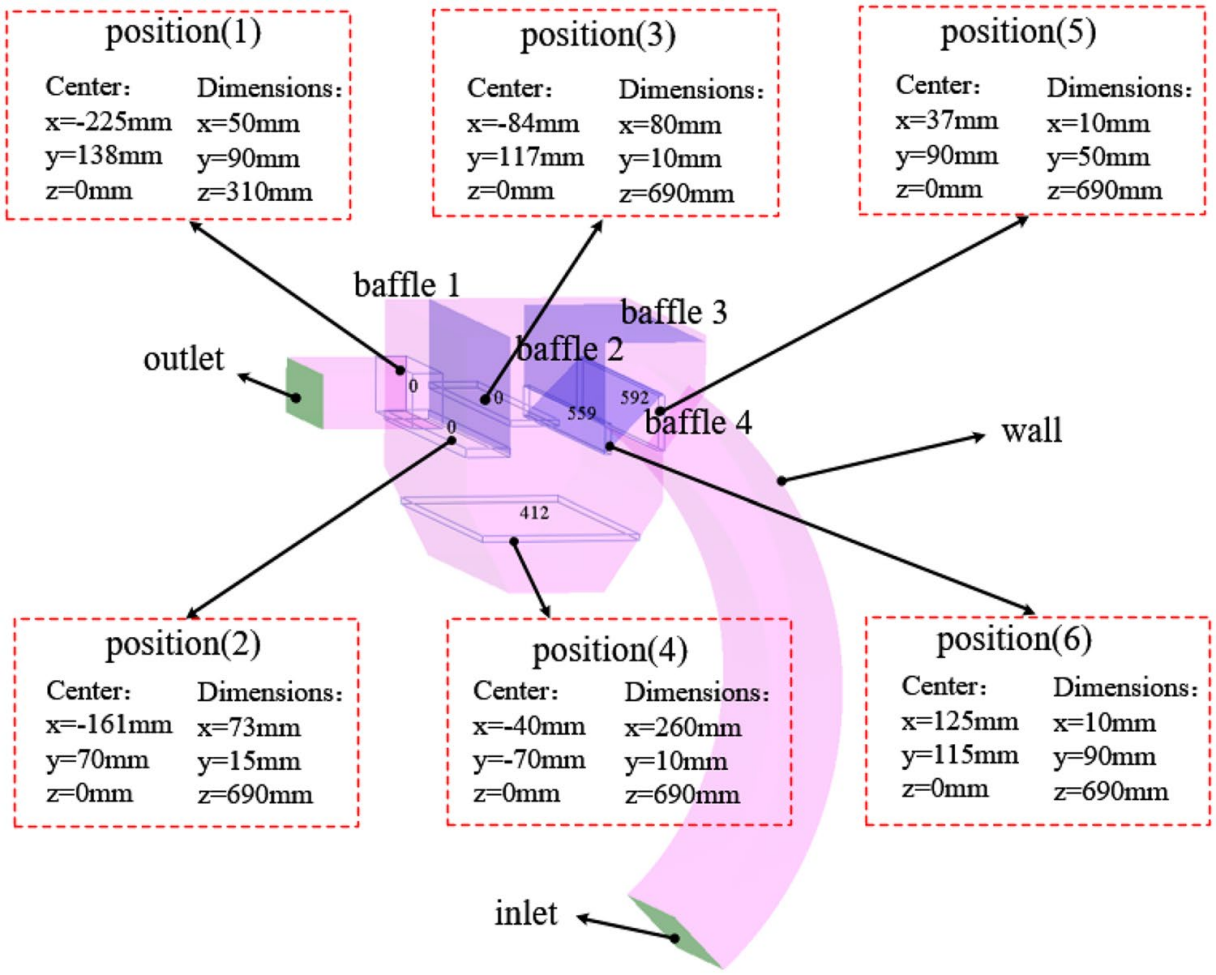
Particle data recording location map.

Regarding the site of particle count, referring to the calculation method of wheat pellet and mixed biomass pellet recovery efficiency by Xu^24^ and Zhou^44^, in the EDEM software, set the size of the exit pipe near the recovery bin side as Center: x = − 225 mm, y = 138 mm, z = 0 mm, Dimensions: x = 50 mm In this option, the number of steel pellets and scrap particles entering the recycling pipe from the recycling bin will be obtained, taking position (2) to position (6) in turn (as shown in Fig. 12). For this reason, in this section we analyze the effect on the recovery efficiency of steel shot particles and scrap particles at different outlet pressures.

**Figure 13:**
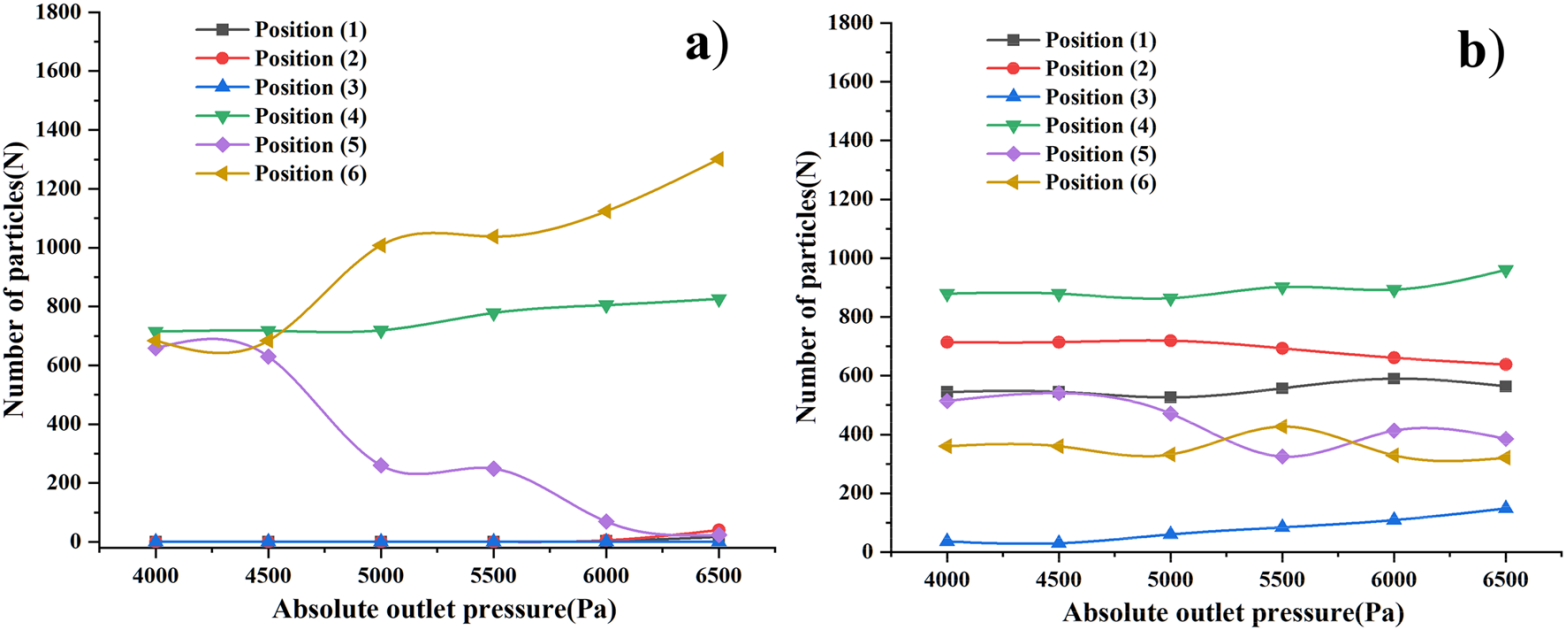
Number of particles in each position at different pressures (**a**) steel shot particles (**b**) waste particles.

As shown in Fig. 12, the 6 positions record the number of particles passing through the position in 1.2 s, for steel shot particles, the total number of steel shot particles entering the recovery bin is the sum of the number of particles at position (5) and position (6), the steel shot entering the bottom of the recovery bin can only be smoothly recovered and enter the next shot blasting cycle processing (Fig. 13).

For waste particles, the total number of steel shot particles entering the recycling bin is the sum of the number of particles in position (5) and position (6), the waste particles entering position (4) does not mean that the waste particles directly enter the shot blasting cycle processing, the particles passing through position (4) can be recycled again under the airflow transport inside the device, the particles entering position (3) will enter the relatively confined environment between baffle 1 and baffle 2 The particles entering position (3) will enter the relatively closed environment between baffle 1 and baffle 2, and the particles will circulate in this area under the action of circulating airflow. The particles that can be discharged smoothly from the recovery bin need to pass through position (2) first. After passing through position (2), the waste particles make an upward movement and the exit pipe transforms itself into a vacuum cleaner similar to a vacuum cleaner under the action of negative pressure for the recovery of waste particles. The waste particles passing through position (1) can be smoothly recovered to the special dedusting mechanism for particle recovery, thus reducing the particle dust pollution caused by the atmosphere.

**Figure 14:**
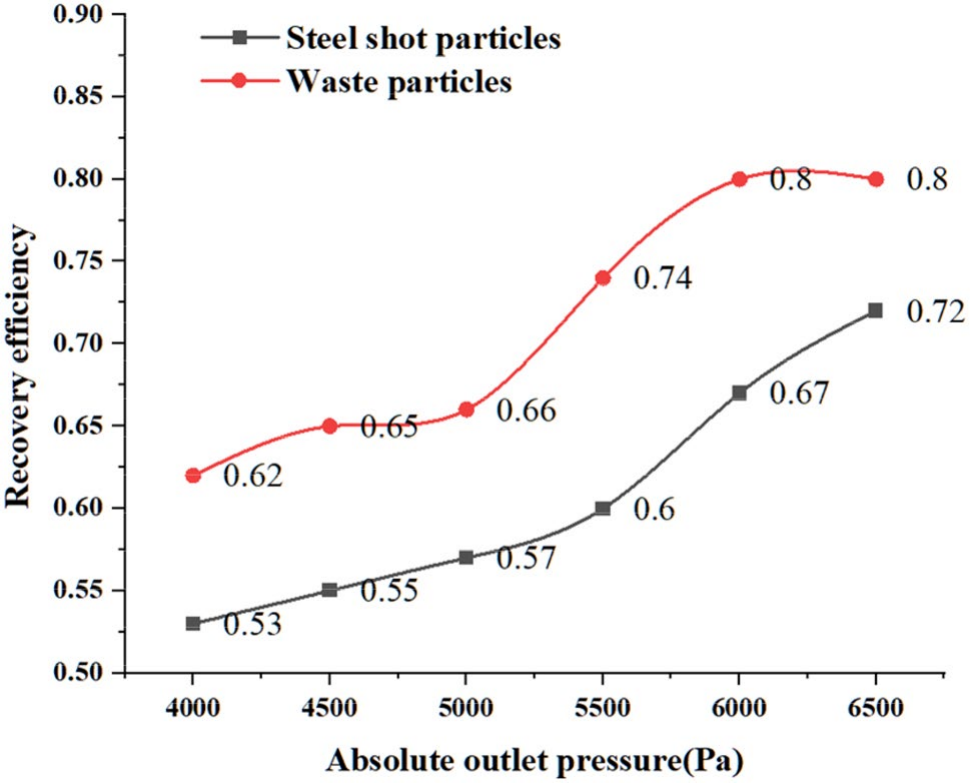
Recovery efficiency of steel shot particles and scrap particles at different absolute pressure outlets.

Regarding the calculation method of particle recovery efficiency, according to two scholars, Xu^24^ and Zhou^44^, who calculated the recovery efficiency and separation efficiency of wheat pellets and mixed biomass pellets, the recovery efficiency of steel pellets is: the difference between the number of pellets at position 4 and position 1 divided by the sum of the number of pellets at position (5) and position (6), and the resulting value is the recovery efficiency of steel pellets. The recovery efficiency of the scrap particles is: the number of particles passing through position (1) divided by the sum of the number of particles in position (5) and position (6), the resulting value is the recovery efficiency of the scrap particles. The recovery efficiency of steel shot particles and scrap particles are calculated to give Fig. 14.

From the particle recovery efficiency graph in Fig. 14, with the increase of the absolute value of the outlet pressure, the recovery efficiency of both kinds of particles increases with the increase of the absolute value of the outlet pressure. The direction of gravity is perpendicular to the direction of airflow, the steel shot particles are more likely to change their original movement path and fall, the speed of steel shot particles into the recovery bin becomes larger, speeding up the recovery process and recovery speed of steel shot; when the absolute value of the outlet pressure is greater than 6000 Pa, under the action of the outlet pressure, a small amount of steel shot particles out of the recovery bin, resulting in the loss of steel shot particles, for this reason, the need to reduce the loss of steel shot because of the outlet pressure The steel shot loss caused by too large outlet pressure.

For waste particles, larger airflow velocity can provide greater conveying force, the Stokes number of waste particles is small, turbulent airflow plays a dominant role in particle distribution, and at the same time, in our study, the internal structure of the recovery bin gives enough time for the waste particles to move with the airflow until the waste particles reach position (1) and exit the recovery unit.

### Experimental verification of outlet pressure on particle recovery efficiency

#### Experiment preparation

**Figure 15:**
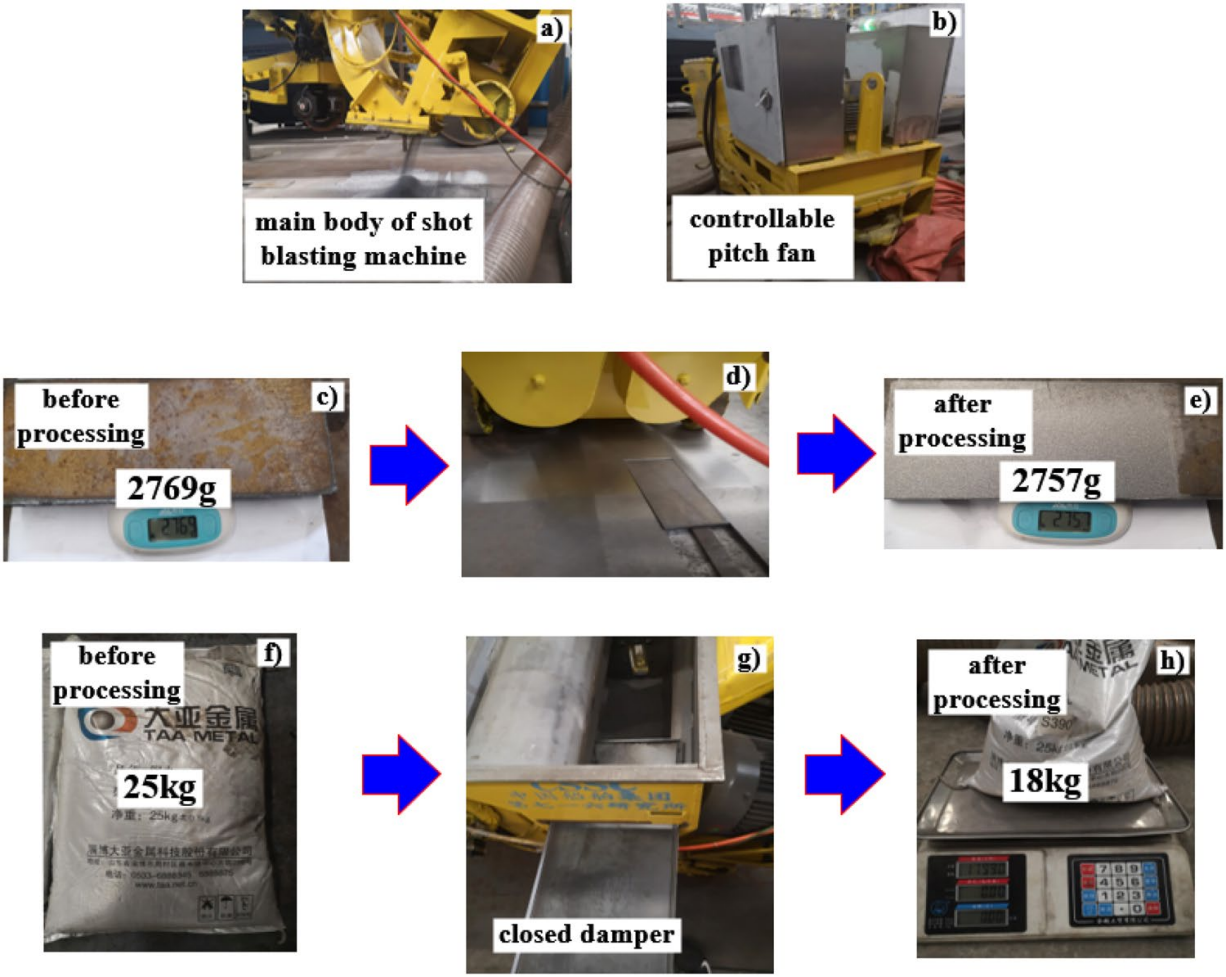
The test device and its schematic diagram.

Outlet pressure on particle recovery efficiency tests were conducted within Zibo Daya Metal Technology JSC, which required the use of the company's ROPW-270 horizontal mobile shot blasting machine, shown in Fig. 15a as the main working part of the blasting machine, and the shot blasting process required this main body to be processed accordingly. Adjustment of the adjustable sub-machine within the waste recovery device of the blast machine in Fig. 15b, the outlet pressure setting, selected − 4000, − 4500, − 5000, − 5500, − 6000 and − 6500 Pa negative outlet pressure for simulation tests to explore the relationship between the outlet pressure and the steel shot particles, waste particles recovery efficiency.

In the enterprise to carry out the marine steel plate under the material, specimen size: 200 mm × 100 mm × 10 mm, the total number of specimens is 12 pieces, to carry out the study between the steel shot particles and scrap particles recovery efficiency.

#### Calculation of recovery efficiency

The total amount of waste particles is calculated as shown in Fig. 15c–e, the difference between the mass of the specimen before and after processing is the total amount of waste particles, recorded as Q_1_; the diameter of the steel shot particles is 2 mm, 25 kg of steel shot is used as the total amount of steel shot within a processing cycle, recorded as Q_2_; the specimen to be processed is installed into the recess for shot blasting according to the installation method in Fig. 15d, and the original structure of the screen is After 25 kg of steel pellets are processed, the blocked pellets are removed from the baffle, and the waste pellets and steel pellets are sieved using a 1.8 mm diameter screen, and the two pellets are weighed with a weighing device after sieving, and the waste pellets q_1_ and steel pellets q_2_ are recorded. At this time, the recovery efficiency of waste particles*η*_1_ is: (*Q*_1_-*q*_1_)/*Q*_1_, the recovery efficiency of steel shot particles *η*_2_ is: *q*_2_/*Q*_1_, record and calculate the relevant data.

#### Analysis of experimental results

**Figure 16:**
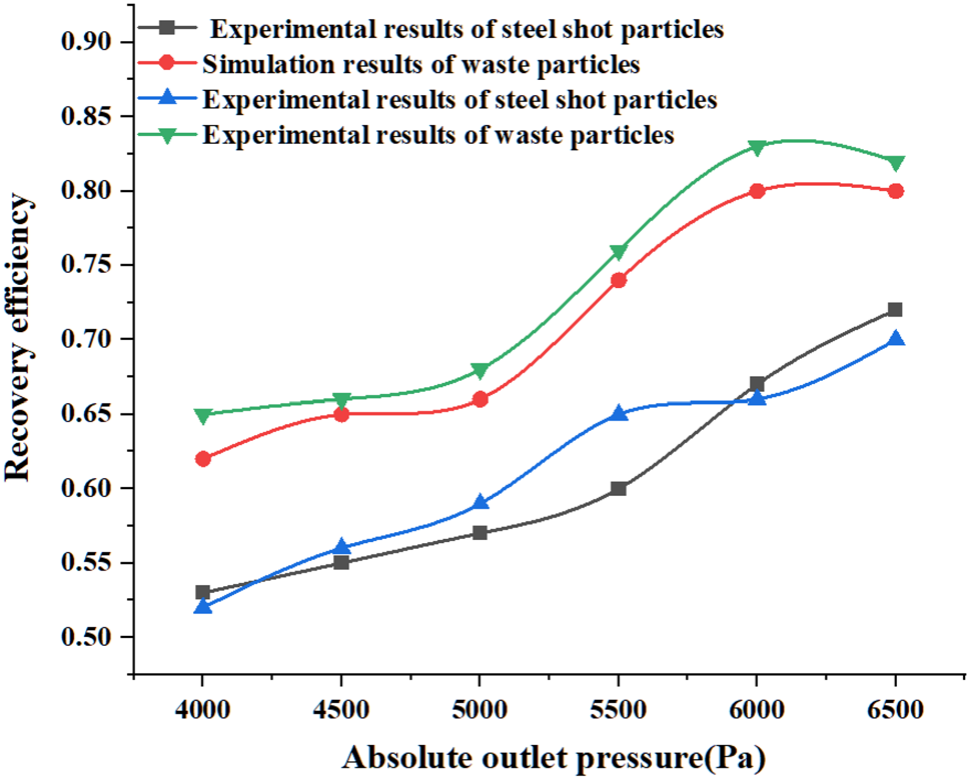
Comparison of recovery efficiency test and simulation.

According to the weighing records of steel shot particles and waste particles before and after processing and the calculation of the recovery efficiency in “Calculation of recovery efficiency”, the resulting recovery efficiency of the two types of particles was compared with the simulation results in “Waste material movement characteristics” and plotted in Fig. 16.

The comparison between the test and the simulation shows that the recovery efficiency of the waste particles increase with the increase of the absolute value of the outlet pressure, and the recovery efficiency of the waste particles measured in the test is slightly higher than that obtained in the simulation, which is caused by the fact that the waste particles are set to 0.2 mm in the simulation, and this value is larger than the waste particles generated on the surface of the specimen after the blasting process in the test. Under the same airflow conveying force, the experimental scrap particles are more susceptible to the airflow conveying force because of their small particle size and small Stokes coefficient, therefore, the recovery efficiency of the experimental scrap particles is slightly higher than that of the simulated scrap particles.

The simulation and test results of steel pellet recovery efficiency are in good agreement, with the absolute value of the outlet pressure increases, the recovery efficiency of steel pellet tends to rise, which is the same trend as the test results, and it can be concluded from Fig. 16 that the recovery efficiency of steel pellet reaches the highest when the outlet pressure is − 6500 Pa. However, under this outlet pressure, according to the analysis result of Fig. 16, a small amount of steel shot particles overcome their own gravity under the conveying force of the airflow and move upward into the outlet pipe, and finally the steel shot particles are discharged from the recycling device and cannot enter the next shot blasting cycle, which causes the waste of steel shot particles and the reduction of shot blasting efficiency.

### Structure optimization

#### Effect of outlet location on pellet recovery efficiency

The previous paper analyzed the fluid distribution and the movement characteristics of steel shot particles and waste particles in the recycling device of the shot blasting machine. In this section, considering the gas phase distribution in the recycling device, the influence of different outlet locations on the particle recovery efficiency will be investigated by changing the outlet pipe and thus the airflow distribution in the device.

**Figure 17:**
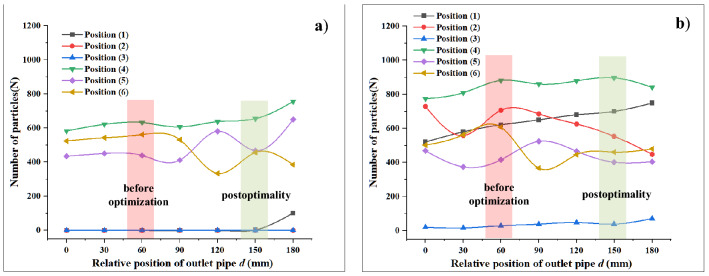
Number of particles passing through the position at different particle generation rates: (**a**) steel shots; (**b**) waste particles.

According to the analysis of the trajectories of steel shot particles and waste particles in sections "Steel pellet trajectory" and "Waste material movement characteristics", the same analysis method as before was used to mesh, import the model and simulate the gas–solid two-phase flow for the outlet pipe model at different locations, and six Grid Bin Groups were pre-set in EDEM software, and all five locations except location (1) were the same as those in section "Effect of outlet pressure on pellet recovery efficiency" The positions are the same as those in Section "Effect of outlet pressure on pellet recovery efficiency". Finally, the particle information is exported in the software, and the information of two kinds of particles is derived as shown in Fig. 17.

**Figure 18:**
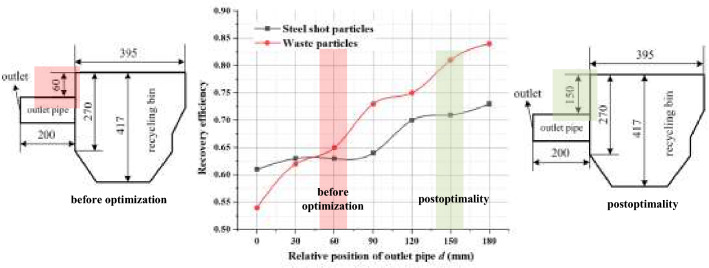
Effect of outlet position on the recovery efficiency of projectile particles and waste particles.

In the same way as the particle recovery efficiency calculation in “Effect of outlet pressure on pellet recovery efficiency”, the number of particles at different locations derived from Fig. 17 was used to calculate the recovery efficiency, and the two particle recovery efficiencies obtained from the calculation were imported into Oringe drawing software to obtain Fig. 18.

From the results in Fig. 18, it can be concluded that when the exit pipe is located at the uppermost part of the left side of the recovery bin (i.e., when *d* = 0), the recovery efficiency of the projectile particles and waste particles is the lowest, and the number of waste particles passing through position (2) is less at this time. The flow of particles is obstructed, and the outlet pipe position produces the smallest value of fluid velocity, which has a smaller conveying force on the waste particles, making the smallest number of particles entering the recycling pipe. With the downward movement of the outlet position, the recovery efficiency of waste particles gradually increases, and when the outlet pipe is located at the bottom, the blocking effect of baffle 1 on the right side of the outlet pipe is the smallest, at this time, the airflow velocity in the recovery bin near the outlet pipe part is the largest, and the conveying effect on waste particles is most obvious in this part.

For steel shot particles, the recovery efficiency of steel shot particles tends to increase during the process of increasing d from 0 to 180 mm, and when d is 180 mm, the recovery efficiency of steel shot particles reaches 72.6%. From the analysis of steel shot particles in Fig. 18, although the highest recovery efficiency at this time, but under this exit pipe position, there are more than 100 projectile particles due to the airflow conveying force is greater than the gravity of the steel shot itself is recovered into the exit pipe, and exclude the exit pipe, under this exit position, not only caused the amount of steel shot particles depletion, but also due to its fan caused by the loss of energy, so the exit position d is 150 mm is the best recovery position, at this time, the recovery efficiency of waste particles is 81.3%, the recovery efficiency of steel shot particles is 70.5%, compared with the recovery efficiency before optimization, the recovery efficiency of steel shot particles increased by 10%, waste particles recovery efficiency increased by 18.9%.

**Figure 19:**
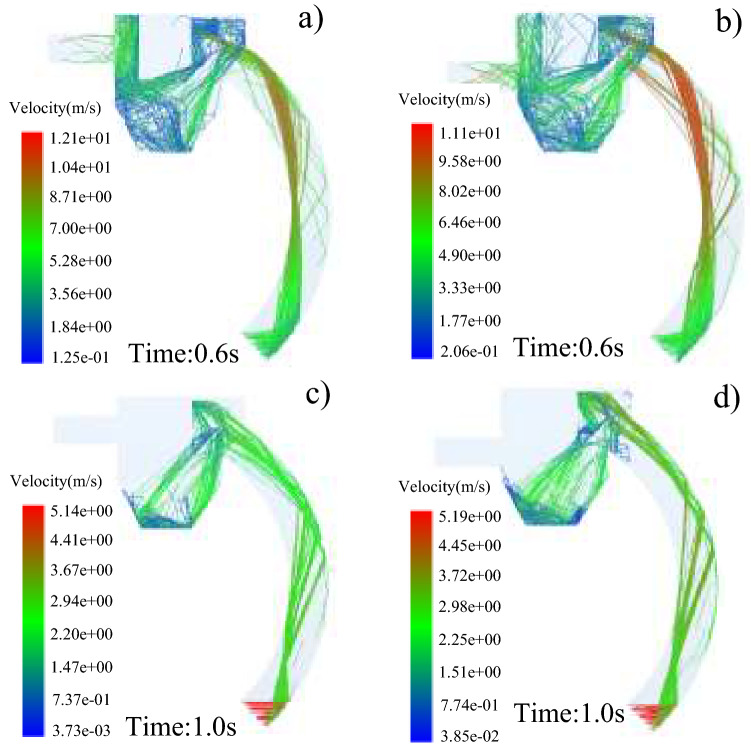
Comparison of outlet pipeline position optimization before and after: (**a**) distribution of waste particle velocity before optimization. (**b**) Distribution of waste particle velocity after optimization. (**c**) Velocity distribution of steel pellets before optimization. (**d**) Velocity distribution of steel pellets after optimization.

The velocity distribution of steel shot particles and waste particles inside the recovery device before and after the optimization of the mobile shot blasting machine is shown in Fig. 19. According to the comparison between Fig. 19a,b, more waste particles enter the recovery pipeline and are excluded from the exit within 0.6 s. It can be seen from the comparison of Fig. 19c,d that within 1.0 s, the number of steel pellets entering the bottom of the recovery chamber increases significantly, which can prove the accuracy of optimization. This conclusion will provide theoretical support and reference for the structural optimization of the horizontal mobile shot blasting machine.

## Conclusion

The shot blasting machine recovery unit is an important component in the operation of a shot blasting machine, and the recovery efficiency of solid particles has a significant impact on its processing efficiency and service life. CFD-DEM simulations and experimental validation were used to investigate the gas–solid two-phase flow characteristics of the shot blast machine recovery unit. The accuracy of the simulations was verified by comparing the results of the coupled calculations with the experimental results obtained in the tests.

- (1) The gas phase distribution, particle motion characteristics and solid particle recovery efficiency inside the shot blasting machine recovery duct and recovery bin at different outlet pressures for steel shot particles and waste particles were investigated. The maximum velocity of the gas phase inside the recovery device increased with the increase of the absolute value of the outlet pressure, and the maximum velocity was 67.59 m/s when the outlet pressure was − 6500 Pa.
- (2) The test and simulation results of the recovery efficiency of steel shot particles and waste particles results show that the recovery efficiency of the two particles increases with the increase of the absolute value of the outlet pressure, when the absolute value of the outlet pressure is greater than 6000 Pa, a small amount of steel shot particles are discharged from the recovery bin under the action of the outlet pressure, resulting in the loss of steel shot particles, so the absolute value of the outlet pressure should not exceed 6000 Pa.
- (3) the blasting machine exit position optimization results show that: the exit position d for 150 mm is the best recovery position, at this time the waste particles recovery efficiency is 81.3%, the steel shot particles recovery efficiency is 70.5%, compared with the recovery efficiency before optimization, the steel shot particles recovery efficiency increased by 10%, waste particles recovery efficiency increased by 18.9%. This value will provide theoretical support and structural optimization reference for the horizontal moving shot blasting machine.

## Supplementary Information

Supplementary Information.

## Data Availability

The datasets used and/or analysed during the current study available from the corresponding author on reasonable request.
